# Genome-Wide Transcriptional Profiling of Skin and Dorsal Root Ganglia after Ultraviolet-B-Induced Inflammation

**DOI:** 10.1371/journal.pone.0093338

**Published:** 2014-04-14

**Authors:** John M. Dawes, Ana Antunes-Martins, James R. Perkins, Kathryn J. Paterson, Marco Sisignano, Ramona Schmid, Werner Rust, Tobias Hildebrandt, Gerd Geisslinger, Christine Orengo, David L. Bennett, Stephen B. McMahon

**Affiliations:** 1 Nuffield Department of Clinical Neurosciences, University of Oxford, John Radcliffe Hospital, Oxford, United Kingdom; 2 Wolfson Centre for Age-Related Disease, King's College London, London, United Kingdom; 3 Department of Structural and Molecular Biology, University College London, London, United Kingdom; 4 Institute of Clinical Pharmacology, Pharmazentrum Frankfurt/Zentrum fuer Arzneimittelforschung, -Entwicklung und -Sicherheit (ZAFES), University Hospital, Goethe-University, Frankfurt am Main, Germany; 5 Boehringer Ingelheim Pharma GmbH & Co. KG, Target Discovery Research Germany, Biberach an der Riß, Germany; Boston Children's Hospital and Harvard Medical School, United States of America

## Abstract

Ultraviolet-B (UVB)-induced inflammation produces a dose-dependent mechanical and thermal hyperalgesia in both humans and rats, most likely via inflammatory mediators acting at the site of injury. Previous work has shown that the gene expression of cytokines and chemokines is positively correlated between species and that these factors can contribute to UVB-induced pain. In order to investigate other potential pain mediators in this model we used RNA-seq to perform genome-wide transcriptional profiling in both human and rat skin at the peak of hyperalgesia. In addition we have also measured transcriptional changes in the L4 and L5 DRG of the rat model. Our data show that UVB irradiation produces a large number of transcriptional changes in the skin: 2186 and 3888 genes are significantly dysregulated in human and rat skin, respectively. The most highly up-regulated genes in human skin feature those encoding cytokines (IL6 and IL24), chemokines (CCL3, CCL20, CXCL1, CXCL2, CXCL3 and CXCL5), the prostanoid synthesising enzyme COX-2 and members of the keratin gene family. Overall there was a strong positive and significant correlation in gene expression between the human and rat (R = 0.8022). In contrast to the skin, only 39 genes were significantly dysregulated in the rat L4 and L5 DRGs, the majority of which had small fold change values. Amongst the most up-regulated genes in DRG were REG3B, CCL2 and VGF. Overall, our data shows that numerous genes were up-regulated in UVB irradiated skin at the peak of hyperalgesia in both human and rats. Many of the top up-regulated genes were cytokines and chemokines, highlighting again their potential as pain mediators. However many other genes were also up-regulated and might play a role in UVB-induced hyperalgesia. In addition, the strong gene expression correlation between species re-emphasises the value of the UVB model as translational tool to study inflammatory pain.

## Introduction

Numerous molecules, produced and released during inflammation either from resident or infiltrating cells, are capable of sensitising nociceptors in the periphery [1]. The prostanoids represent one such group of molecules, which are targeted by current anti-inflammatory pain relief treatments such as the nonsteroidal anti-inflammatory drugs (NSAIDs). However, these widely used analgesics do not provide complete pain relief in many conditions and can cause severe side-effects [2], [3]. Therefore a more complete understanding of what mediators are produced in the context of inflammatory pain may help to develop more efficacious therapies.

Ultraviolet-B (UVB) irradiation of the skin induces a sterile inflammation resulting in a dose-dependent erythema and hypersensitivity to both thermal and mechanical stimulation, and can be used as an inflammatory pain model in humans as well as other mammals [4]–[10]. Irradiation of the skin with UVB is not a painful experience and spontaneous pain does not appear to develop. As a result, sensory changes are confined to the irradiated area suggesting a lack of central sensitisation [5], [6]. In agreement, pain-related behaviour in the rat is not attenuated with N-methyl D-Aspartate (NMDA) receptor blockade and electrophysiological assessment of nociceptors in UVB treated skin show enhanced responses to suprathreshold mechanical stimulation and noxious heat [11]. Therefore it is expected that the sensitisation of peripheral nociceptors, presumably through the action of factors released following inflammation, accounts for the pain-related hypersensitivity seen in this model. As a consequence, this model can be used to screen for previously unrecognised pain mediators. In addition, since this inflammation can be brought about in both man and rodent in a standardised manner, a direct species comparison of the underlying mechanisms which drive UVB-induced pain-related hypersensitivity, and likely other forms of inflammatory pain, can be assessed.

Our previous work has utilised this approach and focused on the regulation of chemokines and cytokines; a group of immune-related factors in which some members have been implicated in modulating pain processing [12], [13]. This work found that many chemokines and cytokines are up-regulated in the skin of the UVB model and that their relative expression changes are comparable between human and rat [14]. On top of this, one highly up-regulated chemokine (C-X-C motif chemokine ligand 5 - CXCL5), with no previous pedigree in pain, was found to play a role in UVB-induced mechanical hypersensitivity [14]. These data confirmed the utility of this approach for identifying new pain mediators and highlight the similarity in the underlying pathophysiology between the two species. In an effort to expand on these results, a genome-wide analysis of transcription in human and rat UVB-treated skin at the peak of pain-related hypersensitivity has been carried out with the use of RNA sequencing (RNA-seq) technology. RNA-seq permits the assessment of expression of all annotated genes but is not limited to these, allowing for novel transcripts and novel areas of transcription to be identified [15]–[17]. Therefore this method offers the possibility of discovering even more previously unrecognised pain mediators as well as allowing for a thorough comparison of UVB-induced gene expression between species.

Differential gene expression (DE) in dorsal root ganglia (DRG) is common to animal models of persistent pain but has not yet been assessed in the UVB model. Therefore, in addition to skin, we also investigated transcriptional changes in L4 and L5 DRG tissue 48 h after UVB irradiation of the plantar paw skin - a time point corresponding to the peak of hypersensitivity.

## Methods

### Ethics Statement

For experiments on human volunteers, approval was obtained from the Ethics committee of King's College London. All subjects provided written consent to take part in the study. All animal experiments were performed in accordance with the United Kingdom Home Office Animals (Scientific Procedures) Act 1986. The Animal Welfare and Ethical Review Body at King's College London approved all procedures (project licence PPL 70/6868). Induction of UVB irradiation was carried out under general anaesthesia and all efforts were taken to minimise suffering of the animals where possible.

### Animals and Human volunteers

For animal experiments male Wistar rats (200–250 g) were used and housed under standard conditions with a 12 hours light/dark cycle with unrestricted access to food and water. Following an initial screen, healthy volunteers with no dermatological conditions (i.e. eczema, dermatitis) and of skin type II or III were permitted into the study.

### Sensory testing

Sensory phenotyping of rats and human volunteers had previously been carried out and is described in Dawes et al, 2011 [14].

### UVB irradiation

Irradiance output of TL01 fluorescent bulbs was calculated using a photometer (IL1400A with SEL240/UVB-1/TD filter, ABLE Instruments and Controls), placed at a predefined distance. This reading was used to calculate the time required to deliver a dose of 1000 mJ/cm^2^ of UVB. Subsequently rats were anesthetised (medetomidine 0.25 mg/kg; ketamine 60 mg/kg, i.p.) and covered using an opaque material, exposing only the plantar surface of the left hind paw. Anesthetised rats were then placed under the fluorescent bulbs at the correct distance so that the exposed skin received the required dose of UVB.

In healthy volunteers, an initial screen was used to calculate the time required for 1 minimal erythemal dose (MED); that is the dose required to produce uniform reddening of a clearly demarcated area 24 hours after irradiation. Following this, all volunteers received 3 MEDs of UVB irradiation to 1 cm^2^ of volar forearm skin using the TL01 florescent bulbs as previously described [14].

### Tissue preparation and RNA extraction

At the peak of UVB-induced hypersensitivity (48 hours) irradiated rats were sacrificed in a CO_2_ chamber and the glabrous skin of the irradiated and un-irradiated plantar hind paws were carefully removed. For L4 and L5 DRG tissue a new cohort of rats were irradiated bilaterally and the DRG samples pooled, naïve rats were used as controls. For qPCR validation experiments, sham animals were anaesthetised but not irradiated. In humans, the irradiated area and an adjacent un-irradiated area was anesthetised using lidocaine and a 3 mm punch biopsy of volar forearm skin was taken. As with the rats, this was also completed 48 hours after irradiation. Following dissection, tissue samples were placed in RNAse-free eppendorf tubes and immediately frozen in liquid nitrogen. For storage, samples were placed at −80°C.

For skin samples total RNA was extracted using a combination of the phenol extraction method and column purification. Briefly, samples were homogenised in Trizol (Invitrogen), mixed with chloroform and spun so that the aqueous liquid phase containing the nucleic acids could be removed. This solution was then placed on Qiagen RNeasy columns and subjected to numerous wash steps to purify the RNA sample. For human skin samples, the RNA used in this study was a subset of the samples previously used for qPCR array analysis [14]. It should be noted that, due to issues with poor RNA integrity of the rat skin samples previously used in the qPCR array cards, RNA used in our RNA-seq experiments was extracted from a separate cohort of animals.

Total RNA from DRG samples was extracted using the miRNeasy kit (Qiagen). All samples were subjected to on-column DNase digestion (Qiagen) to prevent genomic contamination and subsequently analysed using an RNA 6000 nano chip (Agilent Technologies) to confirm sufficient integrity. In addition RNA concentration was measured using a nanodrop (Thermo Scientific).

### RNA Sequencing

RNA samples (500 ng) were used for library preparation using the TruSeq RNA sample preparation kit (Illumina) and subjected to poly-A enrichment. Samples were subsequently sequenced on the Illumina Genome Analyser IIX sequencer. Reads (74 bp unpaired for human skin and 34 bp unpaired end for rat skin and DRG) were mapped to the genome (Rn4 and Hg19 builds for rat and human respectively) with Bowtie allowing for 1 mismatch and unambiguously mapping reads only [18]. Reads mapping to annotated Ensembl genes were counted to estimate gene expression, using the GenomicFeatures R packages. Read counts were normalised to library size. Transcripts with an average of less than 1 read per group in both comparison groups were excluded from further analysis. If this occurred in only one group a value of 1 read was assigned in order to calculate a fold change, necessary for estimation of a numerical fold change and cross-species comparison of expression. DE was calculated with DESeq [19]. Group sample numbers were: Rat skin and DRG, n = 6 irradiated, n = 6 non-irradiated, human skin, n = 5 irradiated and n = 4 non-irradiated. RNA-seq data (fastq files and normalised gene expression matrices) have been made publicly available from the Gene Expression Omnibus (GEO) data base (GSE54413). This data is also available on pain networks [20].

For cross-species correlation analysis of dysregulated genes, orthology data was retrieved from the Ensembl database using the biomaRt R package database [21]. Ensembl orthologues are assigned using the EnsemblCompara GeneTrees method [22]. We considered genes with unique orthologues, or, if more than one possible rat/human orthologue was assigned, the one with highest percentage of sequence identity at the protein level was selected, and the remaining possible orthologues excluded from the analysis.

### Quantitative (q)PCR

qPCR was used to validate the DE measured using RNA-seq. For human and rat skin, we correlated RNA-Seq data with previously published qPCR data for the same RNA samples [14]. Therefore new qPCR experiments were only performed on DRG samples. Prior to qPCR, complementary DNA was prepared using the Superscript III reverse transcription kit (Invitrogen) following the manufacturer's protocol. Samples were prepared in duplicate and added to the Roche LightCycler Master Mix containing SYBR green for the fluorescent detection of real-time changes. These samples were amplified using a Corbett Rotor-Gene 6000 and relative gene expression changes were calculated using the ddCq method normalized against Glyceraldehyde 3-phosphate dehydrogenase (GAPDH). Prior to use primer efficiency was calculated by amplifying a rat cDNA standard of serially diluted amounts. Primers with an efficiency of 0.8–1.2 were used in experiments. All primers were designed using Primer Blast. Primer sequences are shown in Table S1.

### Functional classification of Differentially Expressed Genes

A list of 87 Ensembl gene IDS (human) significantly up-regulated more than four fold in both human and rat skin after UVB irradiation, were classified by “protein class” according to the PANTHER classification system (www.pantherdb.org) [23].

### Statistics

Differential Gene expression for RNA-seq data was calculated using DESeq [19]. False Discovery rate (FDR) was estimated using the method of Benjamini and Hochberg [24] and an FDR threshold of 0.05 was used to control for false discoveries. The same statistical approach was used for qPCR array data following analysis with the R packages ReadqPCR and NormqPCR [25] as previously described [14]. For qPCR data of individual targets, One-way ANOVA with Tukey post-hoc test was carried out on qPCR using the SigmaStat software.

## Results

### UVB-induced gene expression both in human and rat skin

**Figure 1:**
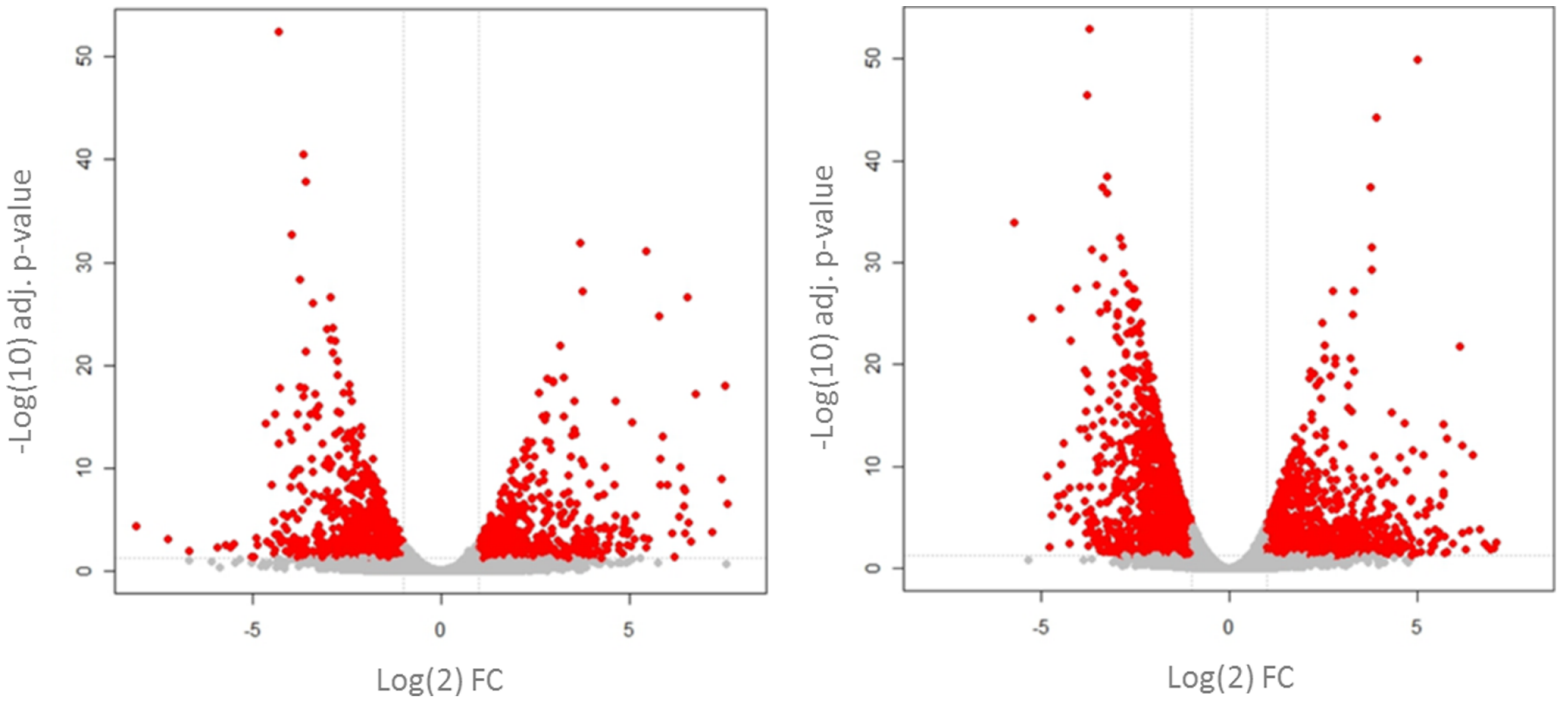
Transcriptional changes in the human and rat UVB model. Volcano plots compare the transcriptional FC (x-axis) versus their level of significance (y-axis). In humans (A) and in rats (B) many genes were significantly dysregulated 48 hours after irradiation in UVB treated skin compared to control. Those genes highlighted in red represent genes which were both statistically significant and at least 2 fold dysregulated in comparison to control. It should be noted that p values are shown on a −log10 scale and FC values on a log 2 scale. FC = UVB/control, human n = 5 irradiated, n = 4 non-irradiated, rat n = 6. Adjusted p-values calculated with FDR.

**Figure 2:**
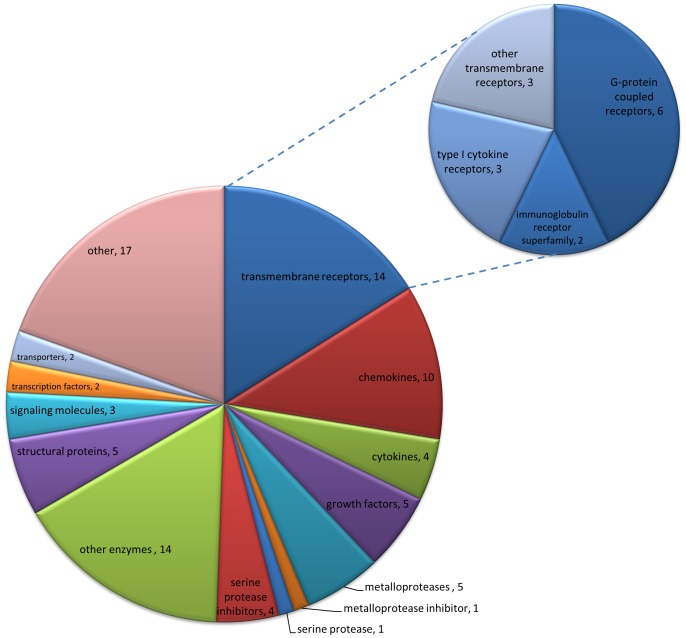
Functional annotation of ‘top hits’. Pie chart with the categorisation of eighty seven genes commonly up-regulated (>4 fold increase in both species, p value <0.05) by “protein category”. Cytokines and chemokines feature prominently (14/87) as well as transmembrane receptors (14/87). Distribution of subcategories of transmembrane receptors is depicted in the small pie chart.

RNA-Seq was used to measure the changes in gene expression caused by UVB 48 hours after irradiation. This time point was selected as it is well established that in both species pain-related hypersensitivity to thermal and mechanical stimuli peaks 48 hours after UVB treatment [4]–[6]. The data are displayed as the fold change (FC) in transcript levels, calculated by dividing the average read counts in the UVB group by those in control (contralateral un-irradiated paw) for each gene. Overall, numerous genes were significantly dysregulated in both the human and rat UVB treated skin (Human 2186 genes, Rat 3888 genes, any FC p<0.05 FDR). In an effort to identify the more robust gene changes, which might be more likely to be contributing to the sensory phenotype, volcano plots were produced for both human (Fig. 1A) and rat (Fig. 1B). Highlighted in red are those genes whose transcript expression was more than two fold different from control and statistically significant. It can be seen that in both species there were a number of genes that, in addition to being highly statistically significant, were also markedly differentially expressed (Fig. 1). It is thought that the abnormal sensory phenotype seen in the UVB model is largely caused by the sensitisation of peripheral nociceptors through the action of inflammatory mediators. Since this hypothesis would require *de novo* or increased expression of such mediators in UVB irradiated skin, we focused on those genes whose transcript levels were increased by UVB treatment in an effort to highlight new putative pain mediators. Using the cut off of >4 fold increase (irradiated vs. non-irradiated skin) in both species and an adjusted p value <0.05, a ‘top hits’ list was created. This consisted of 87 common genes (Table S2) and a functional classification of these by “protein class” is depicted in Figure 2. Amongst these most abundant molecules in both species, cytokines and chemokines represent a sizeable proportion (∼16%). This is in agreement with a known role of the immune system in the UVB model and, interestingly a number of these factors have been shown to contribute to augmented pain signalling [12], [13]. This finding shows that pain mediators were found amongst the ‘top hits’ list, but that many of the other genes in the top hits are yet unknown to the field of pain, and may represent mediators worthy of future investigation.

To gain a better idea of exactly what genes were up-regulated, Table 1 shows the top 50 significantly up-regulated genes in human skin after UVB irradiation. The FC value of the same gene in the rat UVB model is also shown. To gain an idea of the relative abundance of each gene, in the FC column the average number of reads is shown in parenthesis. These values can be compared to the numbers obtained for a selection of commonly used qPCR reference genes in both species (Table 2). On the whole it seems that those genes up-regulated in the human after UVB irradiation were also up-regulated to a similar level in the rat. The top up-regulated gene in the human was Src-homolgy 2 (SH2) Domain containing protein 5 – SH2D5 which had a fold increase of 196.3 and had a FC value of 51.4 in the rat. Although little is known about this specific protein, SH2 domains are known to mediate interactions with phosphorylated tyrosine residues suggesting a role in intracellular signal transduction in receptor tyrosine kinase pathways [26]. In agreement with this, a query for protein-protein interactions using the Pain Networks web resource (painnetworks.org) [27] revealed the growth factor receptor-bound protein 2 (Grb2) and the v-erb-b2 erythroblastic leukaemia viral oncogene homolog 2 (Erbb2, a member of the Epidermal growth factor receptor tyrosine kinases family) as direct SH2D5 interactors. Both Grb2 and Erbb2 also interact with a number of established pain-related genes as listed in the pain genes database [28] and it is therefore possible that SH2D5 is involved in intracellular signalling contributing to pain processing.

**Table 1:** The top 50 UVB-induced up-regulated genes in human and their transcriptional regulation in the rat.

Human gene name (Ensemble ID)	Human FC (number of reads: UVB/Control)	Human p value (FDR)	Rat gene name (Ensemble ID)	Rat FC (number of reads: UVB/Control)	Rat p value (FDR)
SH2D5 (ENSG00000189410)	196.3 (196.3/1.0)	2.4E-07	Sh2d5 (ENSRNOG00000014909)	51.4 (51.4/1.0)	6.18E-08
MMP1∧ (ENSG00000196611)	188.1 (1119.0/5.9)	8.32E-19	Mmp1b (ENSRNOG00000008881)	12.1 (52.4/4.4)	1.31E-06
KRT6C∧∧ (ENSG00000170465)	146.3 (2833.0/19.4)	1.41E-04	LOC683295 (ENSRNOG00000009160)	16.0 (29621/1855.6)	4.49E-05
FOSL1 (ENSG00000175592)	93.1 (768.1/8.2)	2.51E-27	Fosl1 (ENSRNOG00000020552)	10.9 (701.3/64.6)	5.55E-05
IL6 (ENSG00000136244)	89.6 (89.6/1.0)	1.56E-08	Il6 (ENSRNOG00000010278)	88.4 (208.6/2.4)	7.94E-12
MMP3 (ENSG00000149968)	81.5 (476.8/5.9)	6.88E-11	Mmp3 (ENSRNOG00000032626)	13.7 (924.0/67.3)	9.99E-08
CCL20 (ENSG00000115009)	57.0 (57.0/1.0)	1.23E-11	Ccl20 (ENSRNOG00000015992)	44.3 (256.3/5.8)	1.52E-04
S100A9 (ENSG00000163220)	56.7 (18129.5/319.8)	4.44E-09	S100a9 (ENSRNOG00000011483)	22.8 (1692.8/74.3)	7.70E-06
LILRA5∧ (ENSG00000187116)	45.9 (45.9/1.0)	6.71E-04	Lilrc2 (ENSRNOG00000030642)	21.2 (21.2/1.0)	8.90E-05
UUP1 (ENSG00000183696)	43.3 (1046.6/24.2)	4.81E-03	Upp1 (ENSRNOG00000004972)	9.2 (449.9/48.8)	6.23E-03
CXCL5 (ENSG00000163735)	41.8 (92.5/2.2)	5.21E-04	Cxcl5 (ENSRNOG00000002843)	62.1 (62.1/1.0)	3.53E-03
GPR4 (ENSG00000177464)	34.0 (105.6/3.1)	3.31E-15	Gpr4 (ENSRNOG00000016362)	4.8 (188.9/39.2)	2.39E-08
IL1RL1 (ENSG00000115602)	32.5 (218.7/6.7)	1.07E-04	Il1rl1 (ENSRNOG00000014835)	26.6 (464.6/17.5)	1.25E-04
KLK6 (ENSG00000167755)	30.8 (2966.4/96.2)	2.67E-04	Klk6 (ENSRNOG00000031927)	35.8 (2640.7/73.7)	6.82E-12
CXCL1∧ (ENSG00000163739)	29.7 (97.9/3.3)	1.82E-05	Cxcl1 (ENSRNOG00000002802)	32.4 (346.7/10.7)	1.13E-50
CXCL3∧ (ENSG00000163734)	29.5 (48.5/1.6)	9.01E-06	Cxcl3 (ENSRNOG00000028043)	73.4 (73.4/1.0)	3.36E-04
RND1 (ENSG00000172602)	26.5 (26.5/1.0)	7.63E-03	Rnd1 (ENSRNOG00000013621)	10.0 (336.9/33.8)	6.10E-28
KRT16∧ (ENSG00000186832)	26.2 (11665.75/444.9)	3.06E-03	Krt16 (ENSRNOG00000014441)	9.6 (9268.6/969.6)	9.24E-06
KRT6A∧∧ (ENSG00000205420)	25.1 (33093.2/1320.1)	3.10E-17	LOC683295 (ENSRNOG00000009160)	16.0 (29621.0/1855.6)	4.49E-05
IL24 (ENSG00000162892)	24.7 (69.0/2.8)	4.27E-06	Il24 (ENSRNOG00000004470)	14.5 (185.2/12.7)	9.74E-12
HBEGF (ENSG00000113070)	24.4 (910.8/37.3)	4.19E-09	Hbegf (ENSRNOG00000018646)	4.5 (202.6/45.4)	1.31E-04
MMP10 (ENSG00000166670)	24.2 (24.2/1.0)	5.95E-05	Mmp10 (ENSRNOG00000032832)	15.7 (41.4/2.6)	1.14E-05
TNFRSF11B (ENSG00000164761)	22.1 (40.3/1.8)	4.53E-04	Tnfrsf11b (ENSRNOG00000008336)	4.7 (375.7/80.3)	3.03E-07
CLEC4E (ENSG00000166523)	21.5 (21.5/1.0)	8.48E-03	Clec4e (ENSRNOG00000010203)	19.0 (98.7/5.2)	1.13E-06
SERPINE2 (ENSG00000135919)	20.6 (1626.1/78.8)	8.52E-11	Serpine2 (ENSRNOG00000015461)	3.7 (590.3/159.1)	5.65E-06
KIAA1199 (ENSG00000103888)	20.5 (942.0/46.0)	3.76E-05	RGD1305254 (ENSRNOG00000012442)	6.2 (108.8/17.5)	2.48E-02
ADAMTS4 (ENSG00000158859)	20.2 (272.6/13.5)	7.92E-05	Adamts4 (ENSRNOG00000003538)	10.0 (132.6/13.2)	8.39E-09
KRT17 (ENSG00000128422)	17.9 (35150.9/1969.5)	5.78E-08	Krt17 (ENSRNOG00000026371)	19.2 (22785.3/781.0)	1.36E-07
PRSS22 (ENSG00000005001)	17.1 (736.1/43.2)	2.04E-03	Prss22 (ENSRNOG00000039698)	3.6 (40.1/11.1)	6.18E-04
CCL3 (ENSG00000006075)	16.7 (16.7/1.0)	1.08E-03	Ccl3 (ENSRNOG00000011205)	48.8 (213.9/4.4)	7.75E-07
GDF15 (ENSG00000130513)	16.6 (41.9/2.5)	3.65E-02	Gdf15 (ENSRNOG00000019661)	16.5 (92.4/5.6)	3.03E-09
CXCL2∧ (ENSG00000081041)	15.9 (41.5/2.6)	9.13E-05	CXCL2 (ENSRNOG00000002792)	52.8 (192.7/3.7)	6.16E-04
TFPI2 (ENSG00000105825)	15.5 (107.0/6.9)	3.66E-03	Tfpi2 (ENSRNOG00000010513)	29.2 (61.7/2.1)	2.27E-12
KRT6B∧∧ (ENSG00000185479)	15.4 (12390.1/805.1)	6.85E-06	LOC683295 (ENSRNOG00000009160)	16.0 (29621.0/1855.6)	4.49E-05
CD274 (ENSG00000120217)	15.1 (71.2/4.7)	1.61E-05	Cd274 (ENSRNOG00000016112)	5.1 (28.3/5.6)	5.33E-04
OASL (ENSG00000135114)	15.0 (101.5/6.8)	3.30E-02	Oasl (ENSRNOG00000001187)	12.7 (62.9/5.0)	3.41E-02
CCL4 (ENSG00000129277)	14.8 (14.8/1.0)	2.20E-02	Ccl4 (ENSRNOG00000011406)	19.6 (19.6/1.0)	1.48E-03
SELE (ENSG00000007908)	14.4 (295.1/20.5)	6.92E-04	Sele (ENSRNOG00000002723)	3.3 (464.3/139.3)	1.89E-05
TREM1 (ENSG00000124731)	14.4 (14.4/1.0)	4.91E-02	Trem1 (ENSRNOG00000022859)	17.6 (87.5/5.0)	1.03E-03
SOCS3 (ENSG00000184557)	13.7 (735.6/539)	5.79E-28	Socs3 (ENSRNOG00000002946)	15.6 (636.7/40.8)	1.03E-03
SERPINE1 (ENSG00000106366)	12.6 (435.5/34.5)	5.35E-03	Serpine1 (ENSRNOG00000001414)	28.9 (4547.5/157.3)	1.25E-03
TGM2 (ENSG00000198959)	12.5 (111.6/9.0)	1.17E-04	Tgm2 (ENSRNOG00000012956)	4.4 (1071.3/246.0)	2.45E-07
PTGS2 (ENSG00000073756)	12.1 (119.3/9.8)	7.92E-05	Ptgs2 (ENSRNOG00000002525)	7.8 (157.0/20.1)	5.94E-07
ANXA3 (ENSG00000138772)	11.8 (296.1/25.1)	4.42E-14	Anxa3 (ENSRNOG00000002045)	3.8 (233.8/62.1)	1.29E-05
PLPA2G2A (ENSG00000188257)	11.6 (421.4/36.2)	2.40E-03	Pla2g2a (ENSRNOG00000016945)	13.8 (13312.2/965.9)	4.31E-30
TNFRSF12A (ENSG00000006327)	11.6 (450.2/38.9)	1.62E-14	Tnfrsf12a (ENSRNOG00000003546)	4.5 (465.4/102.6)	5.71E-16
TIMP1 (ENSG00000102265)	11.6 (4165.8/360.2)	2.75E-03	Timp1 (ENSRNOG00000010208)	47.9 (8976.6/187.3)	2.57E-04
C13orf33 (ENSG00000102802)	10.9 (996.9/91.7)	7.40E-12	Medag (ENSRNOG00000000906)	3.4 (564.2/164.2)	6.89E-12
PMAIP1 (ENSG00000141682)	10.7 (336.9/31.4)	2.90E-07	Pmaip1 (ENSRNOG00000018770)	12.5 (27.4/2.2)	2.15E-06
LCN2 (ENSG00000148346)	10.7 (294.5/27.5)	1.42E-07	Lcn2 (ENSRNOG00000013973)	14.7 (1020.9/69.5)	3.20E-02

This table shows the top 50 genes whose expression was most increased by UVB treatment in humans compared to control skin 48 hours after irradiation (FDR p<0.05), alongside their respective rat orthologues. Each gene is identified by its gene symbol and ensemble ID. In addition to FC values, the average number of reads for each gene is shown in parenthesis to give a relative idea of abundance. These and FC values have been rounded up to one decimal place. FC = UVB/control, human n = 5 irradiated, 4 non-irradiated, rat n = 6. Adjusted p-values calculated with FDR.

∧ indicates that there were a number of suggested rat orthologues. In this case the gene with the highest target percentage sequence ID at the protein level was used for comparison.

**Table 2:** Fold change and read number for selected reference genes in the human and rat UVB model.

Human gene name (Ensemble ID)	Human FC (number of reads: UVB/Control)	Rat gene name (Ensemble ID)	Rat FC (number of reads: UVB/Control)
GAPDH (ENSG00000111640)	1.7 (7742.5/4632.9)	GAPDH (ENSRNOG00000018630)	1.8 (16.7/9.4)
ACTB (ENSG00000075624)	2.4 (31.832.3/13109.4)	ACTB (ENSRNOG00000034254)	1.6 (15483.3/9520.3)
HPRT1 (ENSG00000165704)	1.6 (72.5/45.2)	HPRT1 (ENSRNOG00000031367)	1.0 (235.4/230.5)
B2M (ENSG00000166710)	−1.1 (7284.3/7641.6)	B2M (ENSRNOG00000017123)	2.4 (2965.4/1265.0)

These 4 selected reference genes are shown here so that read counts can be compared to those in the target gene table. Each gene is identified by its gene symbol and ensemble ID. In addition to FC values the average number of reads for each gene is shown in parenthesis to give an idea of relative abundance. These and FC values have been rounded up to one decimal place. FC = UVB/control, human n = 5 irradiated, 4 non-irradiated, rat n = 6. Adjusted p-values calculated with FDR.

In agreement with previous work we again find that numerous chemokines and cytokines are up-regulated in the skin by UVB irradiation [14], [29]. For example Interleukin 6 (IL6) (FC, human: 89.6; rat: 88.4), CCL20 (FC, human: 57.0; rat: 44.3) CXCL5 (FC, human: 41.8; rat: 62.1), interleukin 24 (IL24) (FC, human: 24.7; rat: 14.5), CXCL1 (FC, human: 29.7; rat: 32.4) CXCL3 (FC, human: 29.5; rat: 73.4) CCL3 (FC, human: 16.7; rat: 48.8) CXCL2 (FC, human: 15.9; rat: 52.8) and CCL4 (FC, human: 14.8: rat: 19.6) were all amongst the top 50 up-regulated genes in the human UVB model and were also up-regulated in the rat. Our previous work suggested that the chemokine CXCL5 contributes to UVB-induced mechanical hypersensitivity [14] and therefore it is interesting that when the whole transcriptome is assessed, CXCL5 is the 11^th^ most up-regulated gene in humans and 14^th^ most up-regulated in the rat after UVB treatment. In addition this list of highly up-regulated genes featured the calcium binding protein S100A9, also known as migration inhibitory factor-related protein 14 or calgranulin-B (FC, human: 56.7; rat: 22.8). Alongside its molecular partner S100A8, S100A9 is a marker of a number of inflammatory conditions in humans and has recently been shown to induce pro-inflammatory cytokine secretion *in vitro* [30]. While these data would suggest that S100A9 is pro-nociceptive, *in vivo* studies have shown that the exogenous administration of the C-terminal domain of this protein exerts an anti-hyperalgesic effect [31], [32]. Therefore further investigations of the role of this protein in nociception in our model system would be an interesting avenue to pursue. Many factors have been proposed as being pain mediators [1], however in terms of those more classical pain genes, only CXCL1, IL6 and cyclooxygenase-2 (COX-2), (FC, human: 12.1; rat:7.8) [33], [34] were amongst the most up-regulated genes at the peak of pain-related hypersensitivity in the UVB model. Therefore amid the top 50 (Table 1) were genes with no previously established function in pain. This finding puts forward the possibility that some of these other up-regulated factors might make a stronger contribution to inflammatory hyperalgesia than those more well-known pain genes. Nevertheless it should be noted that many of these dysregulated genes have known roles outside of the sensory system. For example, one of the most prominent groups among the top 50 genes consisted of members of the keratin gene family. This included KRT6 (FC, human: KRT6A- 25.1, KRT6B- 15.4, KRT6C-146.3; rat: 16.0), KRT16 (FC, human: 26.2; rat: 35.8), KRT17 (FC, human: 17.9; rat: 19.2). These factors represent the primary intermediate filament proteins in epithelial cytoskeleton, have important roles in cell stress, apoptosis and wound healing [35] and are presumably linked to the increased epidermal thickness seen following UVB irradiation of the skin [36]. Therefore keratin gene family members may or may not be directly related to nociceptor sensitization.

### Gene expression is similar in human and rat skin: genome-wide transcriptional profiling

**Figure 3:**
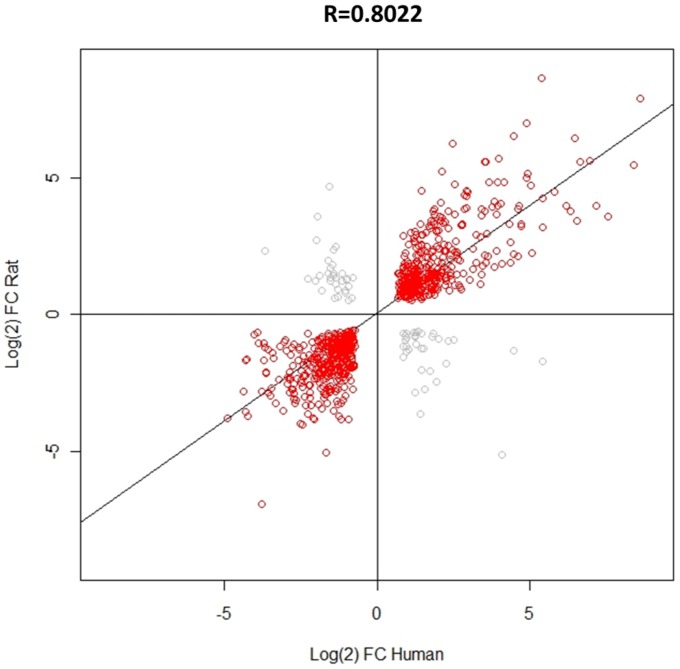
Correlation of human and rat UVB-induced gene expression. Using only those genes which were significantly dysregulated in both human and rat, the Pearson's correlation coefficient (r = 0.8022) showed that, overall there was a positive correlation in FC values. This correlation was highly significant (p<0.001), n = 841 genes in total. A minority of genes did however show an opposite pattern of regulation between species, shown in grey.

Previous data have shown that, in terms of a selection of inflammatory mediators, UVB-induced gene expression is similar between human and rat [14]. Here, RNA-seq has been used to give a more comprehensive comparison of transcriptional regulation between species at the peak of pain-related hypersensitivity, 48 hours after UVB irradiation. Using only those genes which were significantly regulated in both species with a FC>2, 841 in total, FC values were compared and a significant (p<0.001) and strong positive correlation (r = 0.802) was found (Fig. 3). These data suggest that the underlying pathobiology induced by UVB irradiation is similar in both species. Since UVB-induced hypersensitivity is likely the result of inflammatory factors produced in irradiated skin, the underlying mechanisms relating to this process are also comparable between the rat and human. However, despite this overall positive correlation, a few genes were clearly discordant between the two species. That is, they were significantly up-regulated in the rat but significantly down-regulated in the human or vice versa and these are shown in grey.

### Comparison of RNA-seq and qPCR: A validation of gene expression changes

**Figure 4:**
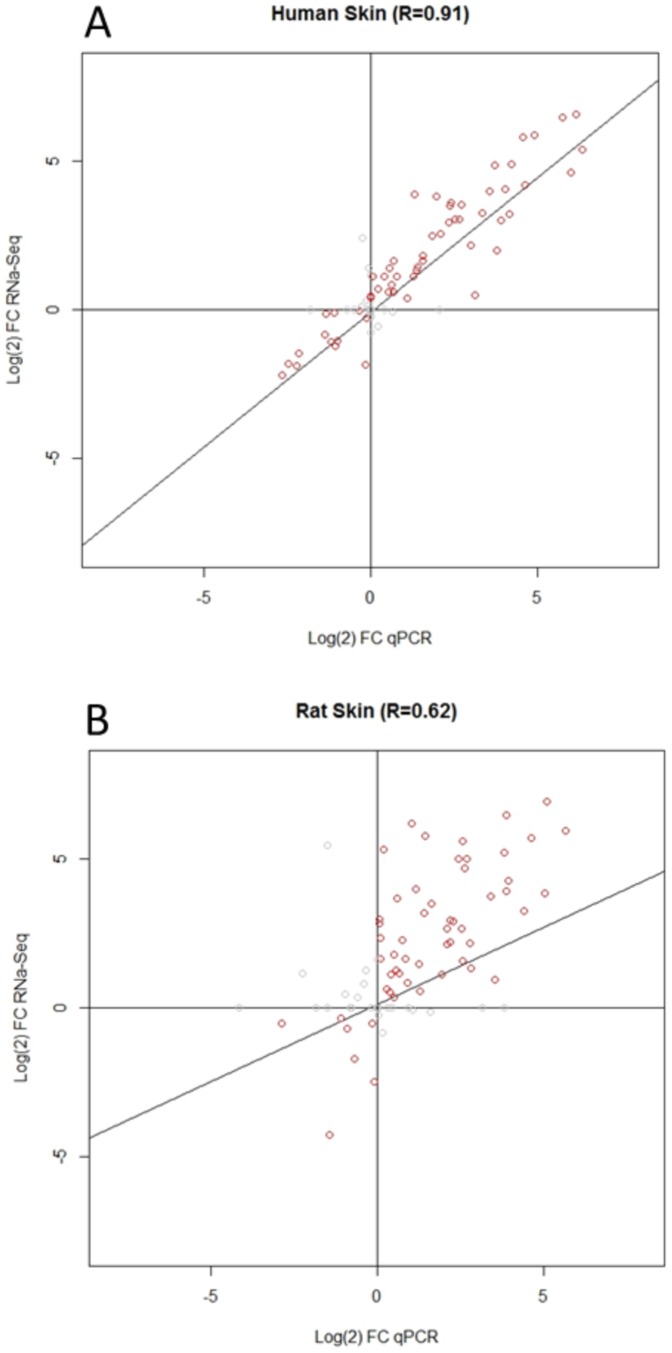
RNA-seq versus qPCR array correlation in the human and rat UVB models. To validate the changes seen using RNA-seq, FC values were correlated against those generated with qPCR array cards (previous data Dawes et al 2011). In both human (A) and rat (B) the Pearson's correlation coefficient (r = 0.911 and 0.620 respectively) showed that there was a positive correlation between the RNA-seq and qPCR array data for both species. These correlations were both highly significant (P<0.001), human n = 92 genes, rat n = 87 genes.

When large scale gene expression studies are carried out, it is necessary to validate changes in transcript levels. This step is normally carried out using the highly sensitive technique of qPCR. Since qPCR analysis of around 90 genes had already been carried out in rat and human skin 48 h after UVB irradiation [14], we aimed to correlate the FC values gained from these results with the FC values obtained for the same genes using RNA-seq. Using the Pearson's correlation coefficient UVB-induced gene expression changes in the human showed a strong positive correlation (r = 0.911) between the qPCR array and RNA-seq data, which was highly significant (p<0.001, Fig. 4A). In the rat this correlation was slightly weaker (r = 0.620), but still highly significant (p<0.001, Fig. 4B). The most likely reason for the weaker correlation observed in rat is that the qPCR results pertain to a distinct cohort of animals from that used for RNA-seq, while the humans samples used for RNA-seq are a subset of those used for qPCR. Taken together these comparisons validate the gene expression changes measured using RNA-seq.

### UVB-induced gene expression changes in the rat DRG

**Figure 5:**
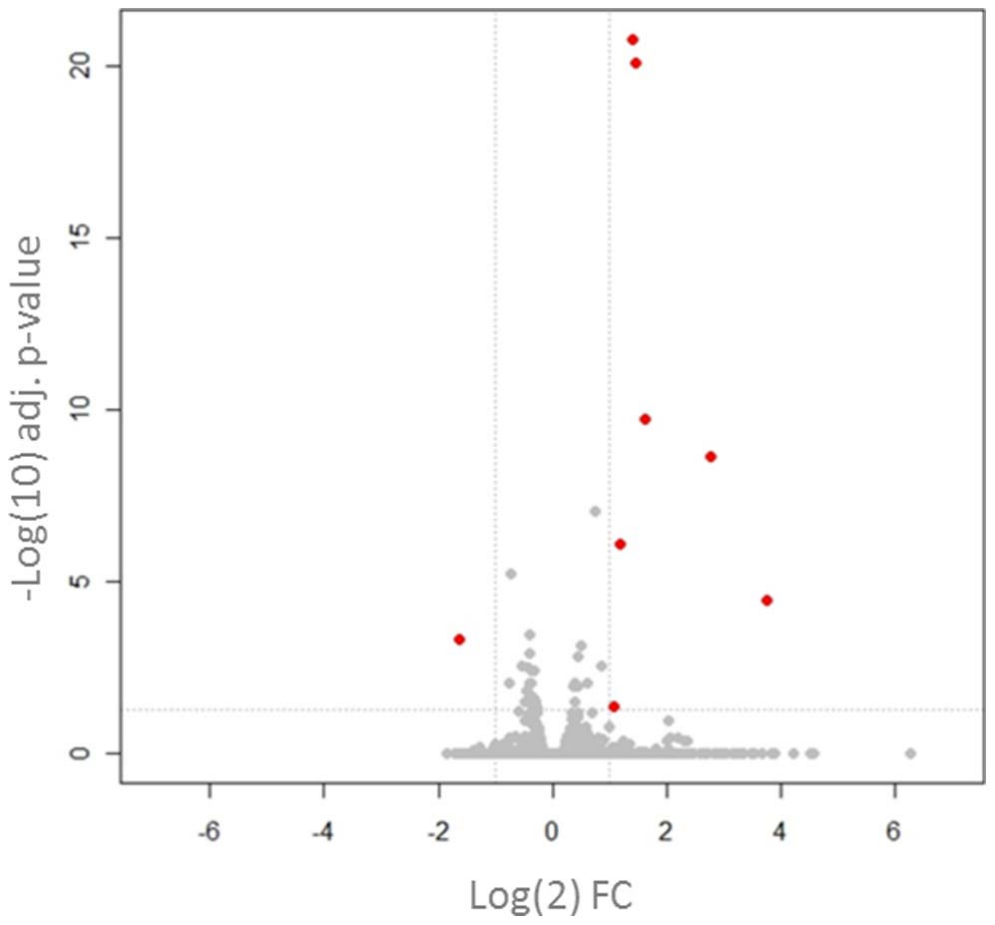
Transcriptional changes in the DRG of the rat UVB model. The volcano plot compares the transcriptional FC (x-axis) of the genes analysed and their level of significance (y-axis). In contrast to the skin, very few significant changes were detected in the DRG 48 hours after UVB irradiation. Those which were significant and had a >2 FC are highlighted in red. FC = UVB/control, n = 8. Adjusted p-values calculated with FDR.

**Figure 6:**
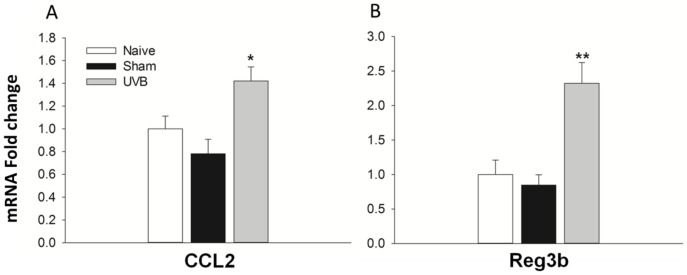
Validation of CCL2 and Reg3b up-regulation in rat DRG with qPCR. Using DRG samples from naive, sham and UVB treated animals the level of CCL2 (A) and Reg3b (B) mRNA was measured using qPCR and normalised against the reference gene GAPDH. For both genes there was a significant increase in their transcript expression in the UVB group compared to sham animals. One-way ANOVA, *p<0.05, **p<0.01, Sham n = 3, Naïve and UVB n = 6.

DRG contain the cell bodies of nociceptors, the neurons which convey pain signalling, as well as other sensory neurons. Because of this, it has been the focus of many gene expression studies in pain models, since transcriptional changes here are likely to render the neurons more or less sensitive e.g. changes in ion channel expression. In addition it can be the site of huge transcriptional change, for example after nerve injury over 1000 genes in the DRG are significantly dysregulated [37]. Since this has not been studied in the DRG after UVB irradiation, RNA-seq was used to measure any potential gene expression changes at the peak of UVB-induced hypersensitivity in the rat DRG. In comparison to skin we found very few significantly dysregulated genes. In total only 39 genes were significantly differentially expressed which is in stark contrast to the 3888 significantly differentially expressed genes found in the skin (Fig. 5). The top 10 most up-regulated genes in the rat DRG are shown in Table 3. In addition, the magnitude of expression changes was substantially less in the DRG as compared to skin. For example, the most up-regulated gene in rat DRG after UVB irradiation was regenerating islet-derived 3 beta (Reg3b) with an FC value of 13.4. Furthermore only 7 genes in total had a greater than 2 fold increase in their expression after UVB irradiation when compared to control (Fig. 5). Despite very few gene expression changes in the DRG, a number of genes in Table 3 have also been found in other models of persistent pain. For example, Reg3b and CCL2 (FC, 2.7) were found to be the two most commonly up-regulated genes in numerous rodent experimental pain models, including nerve injury and chronic inflammation [38]. In addition in the same literature review study, VGF and ATF3 were also found to be up-regulated in 7 and 4 independent experiments, respectively, within the DRG. And both CCL2 and VGF have both been directly implicated in modulating pain processing [39]–[41]. Therefore, despite very little gene expression dysregulation, the DRG might still produce factors important for UVB-induced pain-related hypersensitivity. Since gene expression changes in the DRG after UVB irradiation have not previously been measured, validation of the up-regulation of both Reg3b and CCL2 was confirmed by qPCR (Fig. 6 A, B). When comparing expression levels between naïve and sham treatment groups (animals anaesthetised but not irradiated) no difference was found for both Reg3b and CCL2. However when levels were compared to animals in the UVB treated group a significant increase in the relative expression of both factors was found, confirming the RNA-seq data on UVB-induced changes in the rat DRG.

**Table 3:** Top 10 up-regulated genes in the rat DRG 48 hours after UVB irradiation of the hind paw.

Rat gene name (Ensemble ID)	FC (number of reads: UVB/Control)	P value (FDR)
REG3B (ENSRNOG00000006151)	13.4 (59.3/4.4)	3.30E-05
Protein FAM150B (ENSRNOG00000005154)	6.8 (21.7/3.2)	2.18E-09
Adamts8 (ENSRNOG00000005574)	3.1 (142.0/46.3)	1.74E-10
VGF (ENSRNOG00000001416)	2.7 (136.2/50.0)	7.5E-21
CCL2 (ENSRNOG00000007159)	2.6 (155.1/58.7)	5.79E-06
OSMR (ENSRNOG00000033192)	2.3 (128.5/56.3)	2.52E-06
ATF3 (ENSRNOG00000003745)	2.1 (56.3/26.9)	0.0437
CYSLTR2 (ENSRNOG00000015042)	1.8 (100.9/55.7)	0.0027
NPTX2 (ENSRNOG00000001006)	1.7 (241.8/144.6)	8.65E-08
JAK2 (ENSRNOG00000015547)	1.5 (191.0/125.4)	0.0090

This table shows the top 10 genes whose expression was most increased by UVB treatment in rat DRG compared to control samples. Each gene is identified by its gene symbol and ensemble ID. In addition to FC values the average number of reads for each gene is shown in parenthesis to give a relative idea of abundance. These and FC values have been rounded up to one decimal place. FC = UVB/control, rat n = 6. Adjusted p-values calculated with FDR.

## Discussion

UVB irradiation of either human or rat skin produces a localised sterile inflammatory response which results in comparable levels of thermal and mechanical pain-related hypersensitivity [4], [5], [14]. Unlike most pain models in humans, where the substance applied drives the pain sensation, UVB irradiation, at the dose administered, is not painful. Instead, UVB induces pathobiological changes in the skin resulting in inflammation and hyperalgesia [42]. Subsequent to this, hypersensitivity to sensory stimuli occurs which is likely due to the local release of inflammatory products in the irradiated skin which act to sensitise peripheral nociceptors. Therefore the UVB model can be used to screen for putative peripheral pain mediators in humans. Additionally, the ability to use this model in both humans and rats in a standardised manner, allows for a direct species comparison of the mechanisms which underlie the biological response to an injurious stimulus which produces pain-related hypersensitivity.

To measure transcriptional changes in the UVB skin versus control, RNA-seq was used. This analysis was performed at the peak of UVB-induced hypersensitivity in both species [5], [6] and numerous genes were significantly differentially expressed. Volcano plots highlighted the fact that there were a number of genes which were both highly statistically significant and greatly dysregulated in UVB skin as compared to control. By focusing on only those genes which were significantly more than 4 fold increased in both species a list of common ‘top hits’ was created. When these genes were categorised according to their “protein class”, prominent categories were cytokines and chemokines thus validating the initial approach of focusing on immune-related factors [14]. When genes were ranked by FC in humans, the cytokine IL6 was one of the most up-regulated genes overall. Similar results for IL6 have been found on both a protein and mRNA basis in the UVB model [14], [29] and this cytokine can induce a dose-dependent mechanical hypersensitivity when injected into the paw of naïve rats [43]. Moreover it has been suggested that IL6 can act as a peripheral pain mediator in a model of arthritis [44]; indicating that it could have the same role in UVB treated skin. We also find that there is a significant increase in the expression of the enzyme prostaglandin-endoperoxidase synthase 2 (PTGS2) also known as COX-2 in both species. Since it is known that both in the human and rat, NSAIDs when given systemically or locally, can attenuate UVB-induced pain-related hypersensitivity [4], [45], [46], this finding demonstrates that measuring transcript expression can be used to find genes involved in mediating hyperalgesia.

Previous work using qPCR arrays had found that a number of chemokines were up-regulated in the UVB model [14]. Among the top up-regulated factors in the human (which accounted for 7 of the top 50 genes overall) were the chemokines CCL20, CXCL5, CXCL1, CXCL3, CCL3, CXCL2 and CCL4. In our previous work, CXCL5 was the most up-regulated gene in both human and rat out of a total of 92 genes measured. Furthermore, our functional studies established its contribution to UVB-induced mechanical pain-related hypersensitivity. In accordance with this study, our RNA-seq data revealed that CXCL5 is up-regulated in both species and despite being ranked against the whole transcriptome, CXCL5 remained as one of the most up-regulated transcripts when compared to levels in control skin in both species. CXCL1 and CXCL2, both of which have been implicated in pain [47]–[49], act through CXCR2, the same receptor as CXCL5, suggesting this as an important pathway in UVB-induced pain. The proalgesic action of CXCL1, 2 and 5 is most likely mediated through immune cell recruitment, however CCL3, another highly up-regulated chemokine has been shown to act directly on sensory neurons in vitro by sensitising the activity of the transient receptor potential cation channel subfamily V member 1 (TRPV1) [50]. When injected into naïve mice, this chemokine causes thermal pain-related hypersensitivity [50] and therefore might contribute to this phenomenon in the UVB model. It is also possible that CCL4 has a role in UVB-induced pain since it has recently been shown to act as a peripheral pain mediator after nerve injury [51]. It is notable that the majority of chemokines highly up-regulated in human UVB skin at the peak of pain-related hypersensitivity have been implicated in pain and it will be interesting to investigate whether or not CXCL3 and CCL20 have similar roles. In the current study the use of RNA-seq enabled us to interrogate the whole transcriptome, rather than limiting ourselves to a pre-selected set of genes. Therefore, this genome-wide approach hints at novel roles in pain for some of the other highly up-regulated genes. It should be noted that both resident and invading inflammatory cells contribute to the overall transcriptional profile of UVB irradiated tissues. Gene up-regulation may be the result of active transcriptional regulation in resident or invading cells, or merely constitutive expression reflecting the presence of a new cell type. In this study, Inflammatory cells are likely to make a strong contribution to the expression data since a sizeable proportion of dysregulated genes are immune-related. Conversely resident cells can produce a variety of these factors. For instance keratinocytes and endothelial cells can produce the chemokines CXCL1, 2, 5 and 8 [52], [53] – all of which were up-regulated here. It is also likely that keratinocytes are responsible for the production of keratin gene family members [54]. The chemotactic factors produced by resident cells will recruit inflammatory cells which in turn augment the expression of immune-related genes. For example neutrophils and macrophages can produce cytokines such as IL6 and IL1B [55].

In addition to identifying potential new pain mediators, this study also aimed to more thoroughly compare the transcriptional changes induced by UVB irradiation between the human and the rat. The doses of UVB used here induced a very similar level of thermal and mechanical pain-related hypersensitivity, as well as erythema, used here as a measure of inflammation, in both species [14]. The similarity in the degree of sensory change was mirrored in this this study by the similarity in gene expression changes. We found a highly significant and strong positive correlation between the species, indicating that the same genes were differentially regulated in the human and rat at the peak of UVB-induced pain. Species differences have been blamed for the lack of translation of pain targets found in rodent models to the clinical setting [56]. However here we find that when a standardised injurious stimulus is given to both humans and rats a similar biological response occurs, resulting in a similar level of pain-related hypersensitivity.

Despite a strong overall correlation in the level of transcript expression, there were a small number of genes whose regulation was clearly discordant between species (i.e. significantly up-regulated in one species and down-regulated in the other). One possibility may be inaccuracies in orthologue assignment, which would mean that potentially functionally distinct genes were correlated against each other. On the other hand this may be a true reflection of transcriptional events that are not common between species. Additional work would be needed to assess this further, but the high degree of overlap between the species, provides a large number of putative pain candidates. These represent appealing targets for validation that would be amenable to functional validation in the rat with direct translational implications.

As mentioned, it is likely that the pain-related hypersensitivity experienced following UVB irradiation is the result of the peripheral mechanisms. For example, in the rat, electrophysiological studies have shown that different subsets of nociceptive fibres develop heighten responses to either thermal or mechanical stimuli [11]. This notion is further supported by the fact that sensory modifications are confined to the irradiated area suggesting that there is no central sensitisation [5], [6]. There are a number of mechanisms that can contribute to the sensitisation of peripheral nociceptors and these can be broadly separated into translational and post-translational changes within the neuron. Post-translational changes are most likely the result of inflammatory mediators which induce the phosphorylation or increased membrane trafficking of channels important in the transduction and transmission of nociceptive information [57]. However gene transcription and subsequent translation of new proteins in the DRG can also act to sensitise sensory neurons. For example nerve growth factor (NGF), which is a pain mediator known to be released in inflamed peripheral tissues [58], may induce some of its algogenic effects via the increased transcription of sodium channels in the DRG [59], [60]. Transcriptional changes have been measured in the DRG in other inflammatory pain models [61] but had not yet been carried out in the UVB model. Using RNA-seq we observed high biological variability across individuals, which led to a very limited number of transcriptional changes reaching significance. Overall, only 39 genes were deemed significantly dysregulated in the DRG at the peak of UVB-induced pain-related hypersensitivity. Importantly, the magnitude of FC for the majority of the significant genes was very small, with only seven genes having a FC>2. Nevertheless, the top candidate detected is Reg3b was the most up-regulated. Although it is not clear as to what Reg3b's role in the DRG is, a meta-analysis of gene expression studies in persistent pain models found that this gene was one of the most commonly up-regulated in both rat and mouse [38]. Other genes commonly dysregulated in pain models, and which have been directly implicated in modulating pain processing, include the chemokine CCL2 [40], [41] and the neuropeptide VGF [39]. Both of these were also up-regulated in our rat DRG UVB dataset. One possibility is that these factors act in a juxtacrine fashion via cognate receptors on neuronal cell bodies to sensitise these cells. However in the case of CCL2, it seems that CCR2 is only expressed by DRG neurons following nerve injury [62]. It is true that we measure a small, but significant, increase in the neuronal injury marker activating transcription factor 3 (ATF3) in DRGs from irradiated rats but no increase in CCR2 was seen. Instead work for both CCL2 and VGF have suggested that these factors can act as pain mediators at the level of the spinal cord. For example the intrathecal injection or either VGF or CCL2 can induce pain-related behaviours in rats [39], [40] via the sensitisation of dorsal horn neurons, most likely through their release from the central terminals of nociceptors [39], [63]. The up-regulation of CCL2 and VGF at the level of the DRG would fit with this hypothesis. However if dorsal horn neurons were sensitised an area of secondary pain-related hypersensitivity would be expected, which is absent from the UVB model [5].

Therefore it is likely that the transcriptional changes measured in the DRG at this time point contribute little to UVB-induced pain hypersensitivity seen with this irradiation protocol. Instead these data strengthens the idea that it is mediators produced in the context of inflammation in the skin which, by either acting directly on nociceptors or through the recruitment of immune cells, drive the sensitisation of nociceptors and underpin the pain-related hypersensitivity experienced following UVB irradiation. This notion is further supported by recent lipid profiling data showing that far greater changes were found in the irradiated skin rather than the DRG of the rodent UVB model [64]. It is of course possible that expression changes in the DRG might be more prominent for maintaining pain after its initial peak or following repetitive UVB irradiation which could lead to longer lasting pain states. However using a single stimulus the amount of gene regulation in this tissue is minimal. Another possibility is that pronounced transcriptional changes occur in the “affected” subset of neurons, i.e. nociceptors whose afferents innervate the irradiated hind paw, but cannot be detected at the whole tissue level as they are diluted by “unaffected” cell types. Either way, the lesser changes measured in the DRG do suggest a limited role in this model.

In summary, our data further emphasize the relevance of the UVB model as a highly translational model of inflammatory pain. Moreover, we have expanded on qPCR array data by interrogating the rat and human genome at the genome-wide level using RNA-seq. Our gene expression data again points towards chemokines as being a group of important pain mediators, but also reveals putative novel pain mediators amenable to future validation. In general similar gene expression changes were measured in factors for both the human and rat, strengthening the clinical implications of these findings for future pain research.

### Data Access

http://www.ncbi.nlm.nih.gov/geo/query/acc.cgi?acc=GSE54413

## Supporting Information

Table S1 **Primer sequences used for qPCR, designed using primer BLAST.**
(DOCX)

Table S2 **Fold change, read number and statistical significance for the 87 ‘top hit’ genes.** These genes were identified using a fold increase of >4 and a p<0.05 in both species. A functional classification of these genes by “protein class” is shown in Figure 2.
(DOCX)
