# Supplementary material for: Genome-Wide Transcriptional Profiling of Skin and Dorsal Root Ganglia after Ultraviolet-B-Induced Inflammation
Source: PLoS One. 2014 Apr 14;9(4):e93338. doi: 10.1371/journal.pone.0093338 (PMC3986071; doi:10.1371/journal.pone.0093338)
Supplement: Table S1 — Primer sequences used for qPCR, designed using primer BLAST. (DOCX) [file pone.0093338.s001.docx]

Table S1. Primer sequences used for qPCR, designed using primer BLAST.

| Gene | Forward | Reverse |
| --- | --- | --- |
| CCL2 | TGCTGTCTCAGCCAGATGCAGTTA | TACAGCTTCTTTGGGACACCTGCT |
| Reg3b | TCCATGACCCCACTCTTGGTGGA | CTGCCACAGAATCCGCGGTCTA |
| GAPDH | ATGGGAAGCTGGTCATCAAC | CCACAGTCTTCTGAGTGGCA |
