# Supplementary material for: Genome-Wide Transcriptional Profiling of Skin and Dorsal Root Ganglia after Ultraviolet-B-Induced Inflammation
Source: PLoS One. 2014 Apr 14;9(4):e93338. doi: 10.1371/journal.pone.0093338 (PMC3986071; doi:10.1371/journal.pone.0093338)
Supplement: Table S2 — Fold change, read number and statistical significance for the 87 ‘top hit’ genes. These genes were identified using a fold increase of >4 and a p<0.05 in both species. A functional classification of these genes by “protein class” is shown in Figure 2. (DOCX) [file pone.0093338.s002.docx]

Table S2. Fold change, read number and statistical significance for the 87 ‘top hit’ genes.

| Gene symbol | Ensemble ID: Human | Human FC (number of reads: UVB/Control) | Human p value (FDR) | Ensemble ID: Rat | Rat FC (number of reads: UVB/Control) | Rat p value (FDR) |
| --- | --- | --- | --- | --- | --- | --- |
| SH2D5 | ENSG00000189410 | 196.3 (196.3/1.0) | 2.40E-07 | ENSRNOG00000014909 | 51.4 (51.4/1.0) | 6.18E-08 |
| MMP1 | ENSG00000196611 | 188.1 (1119.0/6.0) | 8.32E-19 | ENSRNOG00000008881 | 12.1 (52.4/4.4) | 1.28E-09 |
| KRT6C | ENSG00000170465 | 146.3 (2839.0/19.4 | 1.41E-04 | ENSRNOG00000009160 | 16.0 (29621.0/1855.6) | 4.49E-05 |
| FOSL1 | ENSG00000175592 | 93.1 (768.1/8.3) | 2.51E-27 | ENSRNOG00000020552 | 10.9 (701.3/64.6) | 5.55E-05 |
| IL6 | ENSG00000136244 | 89.6 (89.6/1.0) | 1.56E-08 | ENSRNOG00000010278 | 88.4 (208.6/2.4) | 7.94E-12 |
| MMP3 | ENSG00000149968 | 81.5 (476.8/5.8) | 6.88E-11 | ENSRNOG00000032626 | 13.7 (9234.0/67.3) | 9.99E-08 |
| CCL20 | ENSG00000115009 | 57.0 (57.0/1.0) | 1.23E-11 | ENSRNOG00000015992 | 44.3 (256.2/5.8) | 1.52E-04 |
| S100A9 | ENSG00000163220 | 56.7 (18129.5/319.8) | 4.44E-09 | ENSRNOG00000011483 | 22.8 (1692.8/74.3) | 7.70E-06 |
| LILRA5 | ENSG00000187116 | 45.9 (45.9/1.0) | 6.71E-04 | ENSRNOG00000030642 | 21.2 (1692.8/74.3) | 8.90E-05 |
| UPP1 | ENSG00000183696 | 43.3 (1046.6/24.2) | 4.81E-03 | ENSRNOG00000004972 | 9.2 (449.9/48.8) | 6.23E-03 |
| CXCL5 | ENSG00000163735 | 41.8 (92.5/2.2) | 5.21E-04 | ENSRNOG00000002843 | 62.1 (62.1/1.0) | 3.53E-03 |
| GPR4 | ENSG00000177464 | 34.0 (105.6/3.1) | 3.31E-15 | ENSRNOG00000016362 | 4.8 (188.9/39.2) | 2.39E-08 |
| IL1RL1 | ENSG00000115602 | 32.5 (218.7/6.7) | 1.07E-04 | ENSRNOG00000014835 | 26.6 (464.6/17.5) | 1.25E-04 |
| KLK6 | ENSG00000167755 | 30.8 (2966.4/96.2) | 2.67E-04 | ENSRNOG00000031927 | 35.8 (2640.773.7) | 6.82E-12 |
| CXCL1 | ENSG00000163739 | 29.7 (97.9/3.3) | 1.82E-05 | ENSRNOG00000028043 | 73.4 (73.4/1.0) | 3.36E-04 |
| CXCL3 | ENSG00000163734 | 29.5 (48.5/1.6) | 9.01E-06 | ENSRNOG00000002802 | 32.4 (346.7/10.7) | 1.13E-50 |
| RND1 | ENSG00000172602 | 26.5 (26.5/1.0) | 7.63E-03 | ENSRNOG00000013621 | 10.0 (336.9/33.8) | 6.10E-28 |
| KRT16 | ENSG00000186832 | 26.2 (11665.8/444.9) | 3.06E-03 | ENSRNOG00000014441 | 9.6 (9268.6/969.6) | 9.24E-06 |
| KRT6A | ENSG00000205420 | 25.1 (33093.2/1320.1) | 3.10E-17 | ENSRNOG00000009160 | 16.0 (29621.0/1855.6) | 4.49E-05 |
| IL24 | ENSG00000162892 | 24.7 (69.0/2.8) | 4.27E-06 | ENSRNOG00000004470 | 14.5 (185.2/12.7) | 9.74E-12 |
| HBEGF | ENSG00000113070 | 24.4 (910.8/37.4) | 4.19E-09 | ENSRNOG00000018646 | 4.5 (202.6/45.4) | 1.31E-04 |
| MMP10 | ENSG00000166670 | 24.2 (24.2/1.0) | 5.95E-05 | ENSRNOG00000032832 | 15.7 (41.4/2.6) | 1.14E-05 |
| TNFRSF11B | ENSG00000164761 | 22.1 (40.3/1.8) | 4.53E-04 | ENSRNOG00000008336 | 4.7 (375.7/80.3) | 3.03E-07 |
| CLEC4E | ENSG00000166523 | 21.5 (21.5/1.0) | 8.48E-03 | ENSRNOG00000010203 | 19.0 (98.7/5.2) | 1.13E-06 |
| KIAA1199 | ENSG00000103888 | 20.5 (942.0/46.0) | 3.76E-05 | ENSRNOG00000012442 | 6.2 (108.8/17.5) | 2.48E-02 |
| ADAMTS4 | ENSG00000158859 | 20.2 (272.6/13.5) | 7.92E-05 | ENSRNOG00000003538 | 10.0 (132.5/13.2) | 8.39E-09 |
| KRT17 | ENSG00000128422 | 17.8 (35150.9/1969.5) | 5.78E-08 | ENSRNOG00000026371 | 29.2 (22785.3/781.0) | 1.36E-07 |
| CCL3 | ENSG00000006075 | 16.7 (16.7/1.0) | 1.08E-03 | ENSRNOG00000011205 | 48.8 (213.9/4.4) | 7.75E-07 |
| GDF15 | ENSG00000130513 | 16.6 (41.9/2.5) | 3.65E-02 | ENSRNOG00000019661 | 16.5 (92.4/5.6) | 3.03E-09 |
| CXCL2 | ENSG00000081041 | 15.9 (41.5/2.6) | 9.13E-05 | ENSRNOG00000002792 | 52.8 (192.7/3.7) | 6.16E-04 |
| TFPI2 | ENSG00000105825 | 15.5 (107.0/6.9) | 3.66E-03 | ENSRNOG00000010513 | 29.2 (61.7/2.1) | 2.27E-12 |
| KRT6B | ENSG00000185479 | 15.4 (12390.1/805.1) | 6.85E-06 | ENSRNOG00000009160 | 16.0 (29621.0/1855.6) | 4.49E-05 |
| CD274 | ENSG00000120217 | 15.1 (71.2/4.7) | 1.61E-05 | ENSRNOG00000016112 | 5.1 (28.3/5.6) | 5.33E-04 |
| OASL | ENSG00000135114 | 15.0 (101.5/6.8) | 3.30E-02 | ENSRNOG00000001187 | 12.7 (62.9/4.5) | 3.41E-02 |
| CCL4 | ENSG00000129277 | 14.7 (14.7/1.0) | 2.20E-02 | ENSRNOG00000011406 | 19.6 (19.6/1.0) | 1.48E-03 |
| TREM1 | ENSG00000124731 | 14.4 (14.4/1.0) | 4.91E-02 | ENSRNOG00000022859 | 17.5 (87.6/5.0) | 1.03E-03 |
| SOCS3 | ENSG00000184557 | 13.7 (735.6/53.8) | 5.79E-28 | ENSRNOG00000002946 | 15.6 (636.7/40.8) | 2.17E-10 |
| SERPINE1 | ENSG00000106366 | 12.6 (435.5/34.5) | 5.35E-03 | ENSRNOG00000001414 | 28.9 (4547.5/157.3) | 1.25E-03 |
| TGM2 | ENSG00000198959 | 12.4 (111.6/9.0) | 1.17E-04 | ENSRNOG00000012956 | 4.4 (1071.3/246.0) | 2.45E-07 |
| PTGS2 | ENSG00000073756 | 12.1 (119.3/9.8) | 7.92E-05 | ENSRNOG00000002525 | 7.8 (157.0/20.1) | 5.94E-07 |
| CCL18 | ENSG00000006074 | 11.7 (215.5/18.4) | 3.01E-17 | ENSRNOG00000011205 | 48.8 (213.9/4.4) | 7.75E-07 |
| PLA2G2A | ENSG00000188257 | 11.6 (421.4/36.2) | 2.40E-03 | ENSRNOG00000016945 | 13.8 (13312.2/965.9) | 4.31E-30 |
| TNFRSF12A | ENSG00000006327 | 11.6 (450.2/38.9) | 1.62E-14 | ENSRNOG00000003546 | 4.5 (465.4/102.6) | 5.71E-16 |
| TIMP1 | ENSG00000102265 | 11.6 (4165.8/360.2) | 2.75E-03 | ENSRNOG00000010208 | 47.9 (8976.6/187.3) | 2.57E-04 |
| PMAIP1 | ENSG00000141682 | 10.7 (336.9/31.4) | 2.90E-07 | ENSRNOG00000018770 | 12.5 (27.4/2.2) | 2.15E-06 |
| LCN2 | ENSG00000148346 | 10.7 (294.5/27.5) | 1.42E-07 | ENSRNOG00000013973 | 14.7 (1020.9/69.5) | 3.20E-02 |
| CCR1 | ENSG00000163823 | 10.3 (116.1/11.2) | 5.48E-03 | ENSRNOG00000006715 | 7.2 (470.0/65.3) | 1.16E-02 |
| IL1B | ENSG00000125538 | 9.6 (48.5/5.1) | 3.39E-03 | ENSRNOG00000004649 | 7.5 (541.2/71.7) | 1.58E-05 |
| NR4A3 | ENSG00000119508 | 8.9 (57.9/6.5) | 4.71E-06 | ENSRNOG00000005964 | 5.4 (104.1/19.4) | 4.12E-06 |
| SH2D2A | ENSG00000027869 | 7.8 (32.2/4.1) | 2.15E-02 | ENSRNOG00000013294 | 18.6 (18.6/1.0) | 1.36E-02 |
| CCL2 | ENSG00000108691 | 7.7 (275.5/35.9) | 2.24E-04 | ENSRNOG00000007159 | 15.1 (1241/82.3) | 5.99E-45 |
| GPRC5A | ENSG00000013588 | 7.7 (727.2/94.9) | 7.92E-05 | ENSRNOG00000008412 | 23.0 (252.2/10.9) | 1.15E-11 |
| HK3 | ENSG00000160883 | 7.6 (48.1/6.3) | 3.52E-02 | ENSRNOG00000026235 | 20.3 (169.4/8.4) | 9.08E-03 |
| CCR7 | ENSG00000126353 | 7.3 (75.5/10.4) | 7.39E-03 | ENSRNOG00000010665 | 20.7 (77.2/3.7) | 4.21E-04 |
| SLC6A14 | ENSG00000087916 | 7.3 (245.7/33.9) | 1.45E-03 | ENSRNOG00000005687 | 14.8 (506.4/34.2) | 2.14E-03 |
| ADAMTS1 | ENSG00000154734 | 7.0 (620.0/89.1) | 6.74E-16 | ENSRNOG00000001607 | 9.7 (722.7/74.2) | 1.08E-25 |
| SLC11A1 | ENSG00000018280 | 6.9 (134.6/19.6) | 2.33E-03 | ENSRNOG00000014956 | 9.9 (93.7/9.5) | 4.87E-02 |
| PLAUR | ENSG00000011422 | 6.7 (216.4/32.3) | 2.72E-10 | ENSRNOG00000037931 | 9.7 (87.7/9.0) | 1.27E-09 |
| RAB20 | ENSG00000139832 | 6.3 (47.0/7.5) | 9.19E-03 | ENSRNOG00000023991 | 4.1 (114.3/28.0) | 1.57E-02 |
| PTGIR | ENSG00000160013 | 6.2 (142.3/23.0) | 3.42E-03 | ENSRNOG00000016756 | 4.9 (92.8/18.8) | 5.14E-09 |
| CYR61 | ENSG00000142871 | 6.1 (1049.5/170.9) | 2.61E-09 | ENSRNOG00000014350 | 4.5 (957.5/211.0) | 4.66E-04 |
| FGF7 | ENSG00000140285 | 5.9 (187.7/31.8) | 5.44E-04 | ENSRNOG00000009425 | 6.4 (219.2/34.2) | 1.05E-04 |
| SRGN | ENSG00000122862 | 5.9 (435.6/74.0) | 4.30E-02 | ENSRNOG00000000394 | 12.6 (583.9/46.4) | 7.99E-03 |
| SCIMP | ENSG00000161929 | 5.9 (19.2/3.3) | 4.32E-02 | ENSRNOG00000037409 | 27.3 (240.3/8.8) | 3.07E-04 |
| P2RY6 | ENSG00000171631 | 5.8 (29.3/5.1) | 1.04E-02 | ENSRNOG00000019270 | 4.9 (208.5/42.2) | 1.53E-02 |
| CCL19 | ENSG00000172724 | 5.6 (428.0/76.4) | 7.90E-03 | ENSRNOG00000015668 | 55.2 (55.2/1.0) | 2.08E-02 |
| TMEM2 | ENSG00000135048 | 5.6 (314.3/56.2) | 6.66E-12 | ENSRNOG00000012782 | 5.3 (632.7/120.5) | 1.93E-08 |
| PHLDA1 | ENSG00000139289 | 5.3 (919.9/172.7) | 3.28E-13 | ENSRNOG00000004019 | 4.1 (462.1/112.5) | 7.28E-06 |
| GJA5 | ENSG00000143140 | 5.3 (65.1/12.2) | 1.86E-02 | ENSRNOG00000017484 | 4.5 (47.0/10.4) | 2.94E-04 |
| SOD2 | ENSG00000112096 | 5.0 (4500.6/899.0) | 6.68E-05 | ENSRNOG00000019048 | 13.8 (4920.0/357.1) | 1.18E-06 |
| MSR1 | ENSG00000038945 | 5.0 (38.8/7.8) | 1.57E-02 | ENSRNOG00000012779 | 8.9 (159.0/17.9) | 1.26E-04 |
| CD68 | ENSG00000129226 | 5.0 (795.6/159.5) | 1.50E-05 | ENSRNOG00000037563 | 5.8 (600.8/103.0) | 6.43E-03 |
| OSMR | ENSG00000145623 | 4.8 (673.2/140.7) | 6.65E-12 | ENSRNOG00000033192 | 5.0 (603.5/121.1) | 9.73E-19 |
| IGFBP4 | ENSG00000141753 | 4.7 (7749.4/1646.5) | 1.69E-03 | ENSRNOG00000010635 | 10.5 (6638.3/629.4) | 1.44E-03 |
| NRG1 | ENSG00000157168 | 4.6 (44.5/9.6) | 2.23E-03 | ENSRNOG00000010392 | 5.0 (215.9/42.8) | 8.11E-14 |
| IL4R | ENSG00000077238 | 4.6 (1145.8/248.7) | 1.30E-12 | ENSRNOG00000015441 | 5.9 (3104.5/529.1) | 1.60E-02 |
| SERPINB9 | ENSG00000170542 | 4.6 (215.6/47.4) | 3.02E-07 | ENSRNOG00000033772 | 5.0 (183.5/36.4) | 9.43E-06 |
| SLFN5 | ENSG00000166750 | 4.5 (75.4/16.8) | 1.44E-03 | ENSRNOG00000037113 | 4.3 (897.8/210.1) | 3.78E-07 |
| ELF3 | ENSG00000163435 | 4.5 (410.7/91.9) | 3.58E-02 | ENSRNOG00000006330 | 10.3 (84.5/8.2) | 3.32E-03 |
| ANGPTL4 | ENSG00000167772 | 4.3 (227.9/52.7) | 8.35E-03 | ENSRNOG00000007545 | 37.5 (1314.5/35.1) | 5.71E-06 |
| PLA2G7 | ENSG00000146070 | 4.3 (54.6/12.6) | 6.10E-03 | ENSRNOG00000025691 | 11.7 (201.4/17.2) | 3.02E-02 |
| SLPI | ENSG00000124107 | 4.3 (1335.3/313.3) | 4.02E-02 | ENSRNOG00000029508 | 17.1 (62.8/3.7) | 3.65E-04 |
| SAMSN1 | ENSG00000155307 | 4.2 (35.1/8.4) | 2.79E-02 | ENSRNOG00000030930 | 10.8 (61.8/5.7) | 6.51E-04 |
| CH25H | ENSG00000138135 | 4.2 (42.7/10.2) | 4.34E-03 | ENSRNOG00000019141 | 12.0 (97.5/8.2) | 2.36E-03 |
| COTL1 | ENSG00000103187 | 4.1 (758.6/187.3) | 7.63E-04 | ENSRNOG00000016257 | 8.4 (2365.6/280.7) | 9.76E-04 |
| ICAM1 | ENSG00000090339 | 4.0 (426.7/105.7) | 2.06E-06 | ENSRNOG00000020679 | 6.8 (996.1/146.6) | 6.10E-28 |
| GFPT2 | ENSG00000131459 | 4.0 (182.3/45.5) | 1.61E-03 | ENSRNOG00000002810 | 5.3 (517.8/97.9) | 4.11E-19 |
